# Astrocytes, Noradrenaline, α1-Adrenoreceptors, and Neuromodulation: Evidence and Unanswered Questions

**DOI:** 10.3389/fncel.2021.645691

**Published:** 2021-02-25

**Authors:** Jérôme Wahis, Matthew G. Holt

**Affiliations:** ^1^Laboratory of Glia Biology, VIB-KU Leuven Center for Brain and Disease Research, Leuven, Belgium; ^2^Department of Neurosciences, KU Leuven, Leuven, Belgium; ^3^Leuven Brain Institute, Leuven, Belgium

**Keywords:** α1-adrenoceptor, α1-adrenergic receptors, noradrenaline (NA), norepinephrine (NE), neuromodulation, astrocyte–neuron interaction, astrocyte calcium

## Abstract

Noradrenaline is a major neuromodulator in the central nervous system (CNS). It is released from varicosities on neuronal efferents, which originate principally from the main noradrenergic nuclei of the brain – the locus coeruleus – and spread throughout the parenchyma. Noradrenaline is released in response to various stimuli and has complex physiological effects, in large part due to the wide diversity of noradrenergic receptors expressed in the brain, which trigger diverse signaling pathways. In general, however, its main effect on CNS function appears to be to increase arousal state. Although the effects of noradrenaline have been researched extensively, the majority of studies have assumed that noradrenaline exerts its effects by acting directly on neurons. However, neurons are not the only cells in the CNS expressing noradrenaline receptors. Astrocytes are responsive to a range of neuromodulators – including noradrenaline. In fact, noradrenaline evokes robust calcium transients in astrocytes across brain regions, through activation of α1-adrenoreceptors. Crucially, astrocytes ensheath neurons at synapses and are known to modulate synaptic activity. Hence, astrocytes are in a key position to relay, or amplify, the effects of noradrenaline on neurons, most notably by modulating inhibitory transmission. Based on a critical appraisal of the current literature, we use this review to argue that a better understanding of astrocyte-mediated noradrenaline signaling is therefore essential, if we are ever to fully understand CNS function. We discuss the emerging concept of astrocyte heterogeneity and speculate on how this might impact the noradrenergic modulation of neuronal circuits. Finally, we outline possible experimental strategies to clearly delineate the role(s) of astrocytes in noradrenergic signaling, and neuromodulation in general, highlighting the urgent need for more specific and flexible experimental tools.

## Introduction

Astrocytes are a major cell type in the central nervous system (CNS), found across brain regions, generally in a non-overlapping fashion (Nimmerjahn and Bergles, 2015). Astrocytes possess many thin membraneous processes, which extend out from the cell body into the parenchyma, where they contact neurons at synapses, physically isolating these structures. This so-called tripartite synapse structure is remarkably common, with up to 90% of mouse cortical synapses being associated with an astrocyte process (Genoud et al., 2006). Not only are astrocytes necessary for synapse formation and maintenance, they have also been shown to perform key roles in the maintenance of local synaptic homeostasis, such as regulating extracellular K^+^ and neurotransmitter levels (Verkhratsky and Nedergaard, 2018). While these important functions are well established, the past decades have seen an upsurge in astrocyte-oriented neuroscience research, fueled largely by the emergence of new technologies (Yu et al., 2020), revealing that astrocytes are not simple housekeeping cells, which passively service the needs of neurons. In fact, numerous studies have shown that astrocytes are actually a dynamic cell type, which respond to a wide range of stimuli, most notably neurotransmitters and neuromodulators, for which they express a large repertoire of receptors (Verkhratsky and Nedergaard, 2018). Astrocytes respond to stimuli in an active fashion, often measured as transient changes in intracellular calcium ([Ca^2+^]_i_) (Rusakov, 2015; Bazargani and Attwell, 2016; Shigetomi et al., 2016), and can in turn release active signaling molecules (gliosignals) (Vardjan and Zorec, 2015), which induce responses in their target cells (Perea and Araque, 2005; Verkhratsky and Nedergaard, 2018). At the synapse, these small neuroactive molecules have been shown to act on both neuronal and glial cell surface receptors to modulate circuit activity and are more commonly referred to as gliotransmitters (Durkee and Araque, 2019). Changes in astrocyte activity can also modulate their K^+^ buffering capacity, with important consequences for local neuronal excitability (Beckner, 2020). Astrocytes are also central players in CNS energy production, through lactate production and secretion, which is influenced by the activation state of the cell (Magistretti and Allaman, 2018). Moreover, astrocyte morphology often changes in response to stimuli, and such changes affect the interactions of astrocytes with neurons at the tripartite synapse (Zhou et al., 2019; Henneberger et al., 2020). Furthermore, astrocytes are coupled into functional networks via gap junctions, allowing crosstalk between otherwise unrelated and relatively distant synapses, a process referred to as lateral regulation of synaptic activity (Covelo and Araque, 2016).

Interestingly, similar effects on CNS circuits have also been attributed to neuromodulators (Pacholko et al., 2020). Indeed, neuromodulators often exert effects on numerous, otherwise unrelated, synapses to change global CNS activity, in response to specific environmental or internal stimuli (Avery and Krichmar, 2017). Over the past several years, there has been accumulating evidence that several neuromodulators may well exert their effects through astrocytes (Ma et al., 2016; Papouin et al., 2017; Robin et al., 2018; Corkrum et al., 2019, 2020; Mu et al., 2019; Wahis et al., 2021b), a subject which is also reviewed in Pacholko et al. (2020). In this review, we focus on noradrenergic neuromodulation through α1-adrenoreceptor (α1-NAR) signaling, as this G Protein Coupled Receptor (GPCR) is highly expressed in astrocytes and is Gq-coupled, leading to intracellular Ca^2+^ release following noradrenaline receptor (NAR) activation.

**FIGURE 1:**
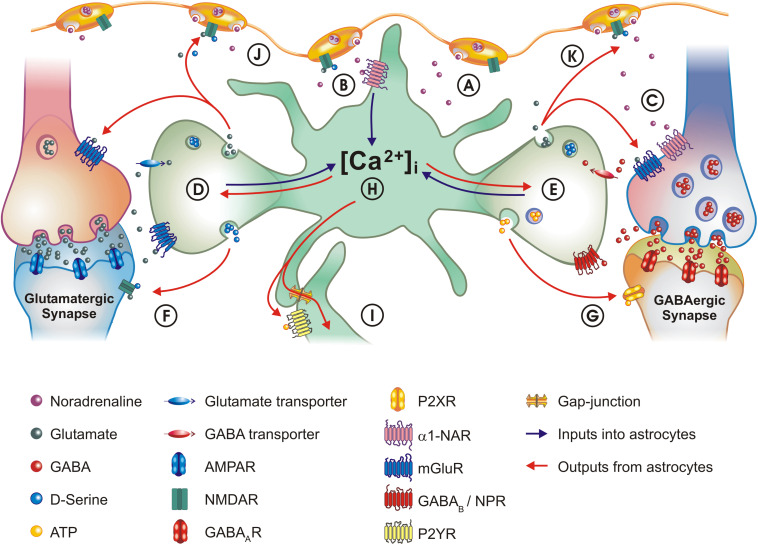
Schematic of astrocyte – neuron interactions and the role of α1-NAR signaling. **(A)** NA is released from LC varicosities into the extracellular space. **(B)** Activation of α1-NAR on astrocytes elicits [Ca^2 +^]_i_ transients. **(C)** GABAergic interneurons also express α1-NAR. **(D)** Astrocytes appear capable of integrating information across signaling modalities: α1-NAR signaling ‘primes’ astrocytes to local glutamatergic synapse activity. **(E)** Astrocytes also integrate activity at GABAergic synapses, through co-activation of neuropeptide receptors (NPR) and GABA_B_ receptors. **(F)** One consequence of astrocyte stimulation is modulation of synaptic activity: one major mechanism by which this occurs is the release of glutamate and D-serine, which activate NMDA receptors, as well as pre-synaptic metabotropic glutamate receptors (mGluR) on neurons. **(G)** Astrocytes can downregulate GABA_A_ currents by releasing ATP which acts on neuronal P2X receptors. **(H)** Astrocytes can transform inhibitory activity at a GABAergic synapse into excitatory signaling at a glutamatergic synapse – a concept known as lateral regulation. **(I)** The concept of lateral regulation also extends to when functionally coupled astrocytes act as bridges, allowing communication between distant synapses. Mechanisms used are either paracrine secretion (local purinergic signaling), or direct transfer of the [Ca^2 +^]_i_ signal (or metabolites) through gap-junctions. **(J)** Integrating synaptic activity with α1-NAR signaling allows astrocytes to participate in the “Glutamate Amplifies Noradrenergic Effects” (GANE) mechanism, in which astrocytes act in a positive feedback loop releasing glutamate, which acts on receptors located in LC axons to stimulate further noradrenaline release. **(K)** A mechanism analogous to “GANE” may also act at GABAergic synapses, with astrocytes transforming GABAergic activity into glutamate release. For purposes of clarity, the size of the different elements are not drawn to scale. In the legend key, R, receptor.

We organize this review according to the sequence of events involved in astrocyte α1-NAR signaling, starting with the release of noradrenaline (NA) and its action on NARs. We then discuss the evidence indicating that α1-NAR activation is not only modulated by prior cellular activation history, but may also ‘prime’ astrocytes to be more sensitive to synaptic inputs. Next, we review the known effects α1-NAR signaling has on astrocyte functions, followed by a discussion of how this then impacts the activity of brain circuits. Furthermore, we advance a hypothesis linking astrocytes and inhibitory neurons as a functional unit in α1-NAR signaling. Key concepts discussed are shown schematically in Figure 1. We also discuss these issues in light of recent advances in our understanding of astrocyte heterogeneity (Pestana et al., 2020), and how this might impact noradrenergic neuromodulation through astrocytes. Finally, we highlight an ensemble of technical approaches that might be relevant in helping us understand, in greater detail, how astrocytes and NA interact to modulate different brain circuits in the CNS.

## NA Release in the CNS

Noradrenaline (also known as norepinephrine) is a catecholamine synthesized from the amino acid tyrosine and is a direct derivative of the neurotransmitter dopamine. It is produced in several groups of neurons, amongst which the brain stem locus coeruleus (LC) is the principal source of noradrenergic afferences in the CNS (Samuels and Szabadi, 2008; Schwarz and Luo, 2015). LC neurons display different firing patterns, consisting either of tonic spikes or phasic bursts of action potentials, which have been associated with the control of wakefulness. Changes in LC firing patterns usually predict changes in behavioral state, with increased tonic firing being correlated with increased arousal, and phasic bursting with the response to salient sensory stimuli (Berridge and Waterhouse, 2003). NA is thought to act on brain function by changing the gain of neuronal circuits, allowing animals to quickly adapt their behavior to changing environmental conditions (Bouret and Sara, 2005; Sara and Bouret, 2012). An interesting feature of noradrenergic transmission is the mode of NA release. Indeed, 20% (or less) of varicosities on monoaminergic (noradrenergic) axons (Vizi et al., 2010), seem to form typical neuronal synapses, while the remainder preferentially face astrocytic processes and release neuromodulators into the extracellular space, which then act as so-called volume transmitters (Figure 1A) (Cohen et al., 1997; Descarries and Mechawar, 2000; Hirase et al., 2014; Fuxe et al., 2015). Crucially, in the case of noradrenergic neurons this figure appears to be lower, with up to 95% of NA varicosities being devoid of synaptic contacts in cortex (Vizi et al., 2010). This morphological evidence is a key component of the hypothesis that astrocytes are important targets of noradrenergic signaling (Stone and Ariano, 1989; Hirase et al., 2014; Fuxe et al., 2015; Pacholko et al., 2020).

## Noradrenergic Receptors in the CNS

Noradrenaline acts on three functional groups of NARs, all of which belong to the GPCR superfamily. α1-NAR are coupled to Gq signaling pathways, α2-NAR to Gi and β1-3-NAR to Gs. Each functional group is further composed of three subclasses, each corresponding to a different gene product: α1A-, α1B-, α1D-NAR; α2A-, α2B-, α2C-NAR; β1-, β2-, β3-NAR. These three functional groups of NAR have different affinities for NA, from highest to lowest: α2 (∼50 nM), α1 (∼300 nM), and β1-3 (∼800 nM), suggesting that cells show different responses depending on the local NA concentration they are exposed to, in combination with the repertoire of NARs expressed (Ramos and Arnsten, 2007; Atzori et al., 2016). In their review, Atzori et al. (2016) build an interesting hypothesis based on this simple starting premise: they argue that activation of the different NARs effectively follows the firing patterns of LC neurons. During sleep, the LC is not firing and no NAR are activated; during quiet wakefulness, increases in both tonic and phasic firing of LC neurons promote activation of α2- and α1-NAR, while β-NAR are only ever activated in “fight or flight” behavioral states, correlated to low phasic firing and a sustained tonic release (Atzori et al., 2016). An interesting *in vivo* imaging study, which reinforces this hypothesis, found that α1-NAR are indeed activated during quiet wakefulness, when significant behavioral stimuli emerge (i.e., when a facial air puff startles the animal). This activation is linked to phasic activity in NAergic neurons. In contrast, β-NAR are only activated during long-lasting aversive stimuli (i.e., repetitive foot-shocks) linked to continual phasic activity of NA axons (Oe et al., 2020). This provides a framework of how NA signaling functions and encodes environmental information relevant for the animal: cells (co)-express various NARs, and their different affinities for NA help the cell distinguish between different levels of extracellular NA, which are a function of the rate and mode of LC axon firing. Interestingly, the cell type that was studied by Oe et al. (2020), and found to respond to activation of both α1- and β-NAR, is the astrocyte. These *in vivo* results confirmed an earlier report showing similar modes of NA signaling in cultured rat astrocytes (Horvat et al., 2016). While these studies clearly demonstrate functional responses of astrocytes to both α1- and β-NAR signaling, other cell types in the CNS also express NARs, which complicates the entire issue of how cell-type specific effects of NA impact on CNS function. Indeed, NAR expression profiles remain a matter of debate and are only being slowly clarified. This is probably due to the difficulties of obtaining reliable antibodies for adrenoreceptors, as well as the shortcomings of genetic models generally used to study this question (in which overexpression of reporter genes, driven by a fragment of the endogenous promoter, might not precisely recapitulate the cellular expression profile of a given NAR; Perez, 2020).

### Astrocytes and α1-NAR

Assessing all the literature on NAR expression patterns in the CNS would constitute a review in itself. As astrocyte Ca^2+^ signaling is central to the modulation of synaptic transmission and neural network excitability (Khakh and McCarthy, 2015; Bazargani and Attwell, 2016), we will focus on the role of α1-NAR signaling in these cells. Indeed, it has been clear for several decades that astrocyte α1-NAR are a prime target of NA (Salm and McCarthy, 1989, 1992; Stone and Ariano, 1989; Shao and Sutin, 1992). α1-NAR are coupled into Gq signaling pathways, which trigger increases in [Ca^2+^]_i_ in astrocytes, following startling stimuli or the activation of LC neurons using electrical stimulation (Figure 1B) (Bekar et al., 2008; Ding et al., 2013; Paukert et al., 2014; Oe et al., 2020). Recent transcriptome studies confirmed mouse astrocytes express transcripts encoding α1A- (*Adra1a*), α1B- (*Adra1b*), and α1D-NAR (*Adra1d*), as well as transcripts for α2- and β-NAR (Cahoy et al., 2008; Hertz et al., 2010; Batiuk et al., 2020). While one of these studies (Hertz et al., 2010) argues for astrocyte-specific expression of α1-NAR, it should be noted that other single cell RNA sequencing studies have found that (inter)neurons also express significant amounts of the various α1-NAR transcripts (Figure 1C) (Zhang et al., 2014; Zeisel et al., 2015). The majority of single cell transcriptome studies, however, are in agreement that α1A-NAR and α1B-NAR appear to be the main α1-NAR subtypes expressed in cortical astrocytes, with α1D-NAR found in much lower amounts, effectively confirming earlier work using transgenic models and radioligand binding (Perez, 2020). Two recent studies independently generated transgenic mouse lines with *LoxP* sites flanking *Adra1a*, allowing conditional deletion of the gene in astrocytes by crossing to appropriate Cre drivers. Both found a loss of calcium transients evoked by enforced locomotion (i.e., a form of startling), or noxious stimuli, in cerebellar (Ye et al., 2020) and spinal cord astrocytes (Kohro et al., 2020), respectively. This is consistent with the transcriptome data from astrocytes isolated from these regions (Anderson et al., 2016; Boisvert et al., 2018). Interestingly, deletion of *Adra1a* was sufficient to result in a loss of NA induced [Ca^2+^]_i_ elevations in both cerebellar and spinal cord astrocytes, despite the expression of *Adra1b* transcripts. This indicates that in these brain regions, the increase in [Ca^2+^]_i_ elicited by α1-NAR activation is mediated principally through the α1A-NAR subtype. Whether this holds true in other brain regions awaits clarification. While these data were all obtained in rodents, the importance of α1-NAR signaling through astrocytes seems evolutionary conserved. Indeed, tyramine and octopamine, the invertebrate equivalents of NA, also signal to astrocyte-like cells in *Drosophila melanogaster.* Moreover, tyramine-octopamine evoked astrocyte [Ca^2+^]_i_ transients are crucial for neuromodulation. Interestingly, the fact that these [Ca^2+^]_i_ transients can be blocked by application of terazosin, an antagonist of vertebrate α1-NAR, highlights the conservation of the basic building blocks used by invertebrates and vertebrates to construct key signaling pathways (Ma et al., 2016). More recently, it was also found that zebrafish α1B-NAR are expressed in radial astrocytes and are essential for NA-mediated responses generated by repeated behavioral (locomotor) failures. Human data are scarce, but indicate that the α1-NAR mRNA expression patterns observed in mouse astrocytes and neurons are essentially conserved (Zhang et al., 2016), while data from the human protein atlas confirm that glial cells are the main cell type expressing *ADRA1A* in human cortex^1^ (Thul et al., 2017). This indicates noradrenergic signaling, through astrocytic α1-NAR, has been conserved throughout evolution, highlighting its importance.

## α1-NAR Triggered Calcium Signaling in Astrocytes: A Switch for Signal Integration?

In this section, we will evaluate evidence indicating that astrocytic [Ca^2+^]_i_ transients evoked by α1-NAR activation can be modulated by interactions with other signaling pathways, and argue that this represents a form of complex signal integration in astrocytes.

### Astrocytes Encode Levels of NA

Phasic release of NA reliably provokes Ca^2+^ transients in astrocytes (Slezak et al., 2019), through α1-NAR signaling (Bekar et al., 2008; Ding et al., 2013; Paukert et al., 2014; Oe et al., 2020). Interestingly, another study found this response is dependent on the degree of NAergic neuron firing. Indeed, Mu et al. (2019) found that zebrafish radial astrocytes encode the number of behavioral (locomotor) failures through successive bouts of NAergic neuron activity and the accumulation of astrocyte [Ca^2+^]_i_ following α1-NAR activation; once a [Ca^2+^]_i_ threshold is reached, GABAergic neurons are then activated by astrocytes, leading to a change in behavioral state. However, the molecular mechanisms by which astrocytes encode the history of α1-NAR activation and modulate GABAergic neuron activity are currently unclear (Mu et al., 2019). However, insights into the possible mechanism(s) can be found in other studies. Nuriya et al. (2017) found that background levels of NA (500 nM) facilitate the astrocyte [Ca^2+^]_i_ response evoked by a short pulse of NA designed to mimic local release from LC varicosities (20 μM). Interestingly, this background ‘priming’ was shown to occur via β-NAR activation and an associated increase in intracellular cAMP levels; in contrast, the astrocyte [Ca^2+^]_i_ response was shown to occur through an α1-NAR-dependent mechanism (Nuriya et al., 2017). Although seemingly counterintuitive, when considering NAR affinities alone, there are a number of plausible explanations accounting for this ‘priming’. β-NAR signaling occurs on a slower timescale than α1-NAR activation (Horvat et al., 2016; Oe et al., 2020). Furthermore, synergistic actions between Gs and Gq-coupled signaling pathways have been reported (Delumeau et al., 1991; Cordeaux and Hill, 2002; Ahuja et al., 2014; Horvat et al., 2016). Hence, low levels of β-NAR signaling may act synergistically with α1-NAR signaling to facilitate astrocyte [Ca^2+^]_i_. Interestingly, β-NAR activation can also increase α1A-NAR expression, probably via post-transcriptional mechanisms, increasing mesenchymal stromal cell sensitivity to NA (Tyurin-Kuzmin et al., 2016). If the same holds true for astrocytes, this could also increase the sensitivity of cells to α1A-NAR activation. Interestingly, the two mechanisms postulated are not mutually exclusive and may act in tandem.

These last studies demonstrate that astrocytes can encode extracellular NA levels, likely through the synergistic effects of different NAR signaling pathways. This would allow astrocytes to encode salient behavioral stimuli (α1-NAR signaling through phasic NA release) with a different weight (encoded in the [Ca^2+^]_i_) depending on the wakefulness state (encoded in the ambient NA levels due to tonic NA release). Importantly, however, it would also incorporate aspects of behavioral history, taking into account the number of prior “startling” stimuli and the associated phasic release of NA.

### Astrocytes Integrate Synaptic Activity Through α1-NAR Signaling

There is also *in vivo* evidence that astrocyte responses to phasic NA release, through α1-NAR signaling, are influenced by local synaptic activity. Paukert et al. (2014) found that Ca^2+^ transients elicited in mouse Bergmann glia by enforced locomotion are fully abolished by an α1-NAR antagonist. However, the transients are also diminished in amplitude when α-amino-3-hydroxy-5-methyl-4-isoxazolepropionic acid (AMPA) and *N*-methyl-D-aspartate (NMDA) receptor antagonists are applied, indicating the involvement of glutamatergic signaling in amplifying astrocyte responses to α1-NAR activation. Furthermore, the authors also found that visual cortex astrocytes do not reliably respond to visual stimuli during periods of quiet wakefulness, but that astrocyte [Ca^2+^]_i_ responses to enforced locomotion (through α1-NAR activation) are amplified if mice are simultaneously presented with a simple visual stimulus (light flash). This indicates that astrocytes integrate sensory inputs dependent on the behavioral state of the animal (Paukert et al., 2014). This result was later confirmed by work demonstrating that visual cortex astrocytes respond to moving visual stimuli in a retinotopic fashion but only when mice are locomoting, an effect which is linked to the presence of NA (Slezak et al., 2019) (although it should be noted that a recent study found contrasting results, with evidence for α1-NAR-mediated [Ca^2+^]_i_ signaling masking astrocyte responses to visual stimuli in mouse; Sonoda et al., 2018). Nonetheless, results similar to those obtained by Paukert et al. (2014) and Slezak et al. (2019) were obtained in zebrafish. Mu et al. (2019) observed that radial astrocytes in the lateral medulla oblongata do not reliably respond to neuronal activity during swimming. However, if NA neurons are optogenetically activated during swimming, radial astrocytes encode swimming-related neuronal activity in the form of robust [Ca^2+^]_i_ transients larger than those evoked by stimulation of NA neurons alone (Mu et al., 2019). This indicates a mechanism, seemingly conserved in vertebrates, in which the noradrenergic system gates, or at least greatly amplifies, the response of astrocytes to local synaptic inputs, through α1-NAR signaling. The mechanisms behind this gating phenomenon remain to be explored, but the data of Paukert et al. (2014) implicate glutamatergic ionotropic receptors. Whether these receptors are expressed alongside α1-NAR on astrocytes, or whether they are expressed independently on other (interacting) cell types, remains unclear at present. It should also be noted that none of the studies performed in mouse visual cortex that we cite unambiguously demonstrate the direct activation of astrocyte α1-NAR to be central to the observed effects. Even if this is the most likely explanation, it remains to be explored in future studies, likely employing the *Adra1a*-floxed mouse lines that were recently reported (Kohro et al., 2020; Ye et al., 2020).

Taken together, these reports clearly indicate that astrocyte responses to α1-NAR are complex, and are capable of integrating information based on recent circuit activity (Figure 1D). It is worth mentioning that signal integration by astrocytes also exists outside the frame of NA signaling. For instance, the [Ca^2+^]_i_ transients induced in mouse cortical astrocytes by GABA released from somatostatin interneurons are potentiated by the co-release of somatostatin that occurs when these neurons are subject to intense activation. However, the activity of parvalbumin interneurons, or somatostatin interneurons when somatostatin receptors are pharmacologically blocked, depresses astrocyte [Ca^2+^]_i_ responses triggered by GABA_B_ receptor activation (Mariotti et al., 2018). Moreover, somatostatin is also able to potentiate the response of cultured striatal astrocytes to α1-NAR activation (Marin et al., 1993). A similar result was observed with another neuropeptide co-released with GABA from interneurons, vasoactive intestinal peptide (VIP). In this study, VIP receptor stimulation in astrocyte cultures could essentially reduce the threshold to evoke [Ca^2+^]_i_ transients through α1-NAR activation, again indicating a synergistic action of the two receptor pathways (Fatatis et al., 1994). This indicates α1-NAR signaling can further be amplified at tripartite synapses involving somatostatin or VIP expressing GABAergic interneurons (Figure 1E, see also section “Astrocytes and α1-NAR: A System Tailored to Regulate Inhibition?”).

So far, it remains unclear how the complex integration of synaptic activity with α1-NAR signaling affects the functional output(s) of vertebrate astrocytes. However, we believe it likely that it allows astrocytes to fine tune the activity of CNS circuits (discussed in sections “α1-NAR Signaling: Effects on Astrocyte Function” and “α1-NAR Signaling in Astrocytes: Relevance for Neuromodulation”).

## α1-NAR Signaling: Effects on Astrocyte Function

α1-NAR signaling has been linked to modulation of many known astrocyte functions, which may or may not involve increases in [Ca^2+^]_i_. However, the majority of these studies did not use cell-type specific manipulations, or were performed using reductionist *in vitro* models, hindering the extrapolation of results to the *in vivo* situation. Nonetheless, they provide interesting perspectives on how α1-NAR signaling could impact on astrocyte functions, with potentially important consequences for α1-NAR mediated neuromodulation.

For instance, activation of α1-NAR has been linked to increases in lactate formation and oxidative metabolism (Subbarao and Hertz, 1991), although most studies attribute α2-and β-NAR activation to metabolic regulation in astrocytes (Hertz et al., 2010), most notably glycogen metabolism (Subbarao and Hertz, 1990; Coggan et al., 2018; Oe et al., 2020). The role of astrocytes in production of lactate and its transport from astrocytes to neurons is an intense area of astrocyte research; it is clearly linked to noradrenergic signaling, but again, is mostly related to β-NAR activation (Magistretti and Allaman, 2018; Zuend et al., 2020). The regulation of astrocyte-mediated energy metabolism by α1-NAR might also be indirect, for instance by its action on glutamate uptake and the consequent increase in its metabolite, α-ketoglutarate (Alexander et al., 1997; Hertz et al., 2010). Interested readers are directed to more extensive recent reviews covering this topic (Dienel and Cruz, 2016; Alberini et al., 2018). The role α1-NARs play in regulating glutamate uptake into astrocytes might be especially relevant when it comes to controlling levels of (local) neuronal activity. Interestingly, increases in the rate of glutamate uptake following NA release seem to be solely linked to α1-NAR activity, while activation of β-NARs is thought to specifically increase the rate of GABA uptake into astrocytes (Hansson and Rönnbäck, 1989, 1992). This hints at potentially opposing roles for these two receptors, with α1-NAR activation decreasing excitatory glutamatergic signaling, and β-NAR decreasing inhibitory GABAergic signaling.

Another key homeostatic function of astrocytes is the maintenance of extracellular potassium levels ([K^+^]_e_). Following action potential firing, astrocytes remove K^+^ from the extracellular space, using both active and passive mechanisms, and redistribute it through the (gap-junction coupled) astrocyte network, facilitating further action potential firing (Beckner, 2020). A recent study found that NA, likely acting through astrocytes, increases the speed at which [K^+^]_e_ reverts to basal levels following neuronal stimulation, and dampens the maximal increase of [K^+^]_e_ reached during high frequency neuronal activity (Wotton et al., 2020). Yet a possible role for α1-NAR in controlling [K^+^]_e_ remains unclear and requires further study. Indeed, most reports indicate a role for β-NAR in increasing K^+^ uptake in astrocytes; in contrast, one study shows that phenylephrine, an α1-NAR agonist, reduces K^+^ uptake in cultured astrocytes (Åkerman et al., 1988; Wotton et al., 2020).

α1-NAR receptors are also involved in controlling the level of gap-junction coupling between astrocytes. α1-NAR activation in cultured astrocytes leads to the phosphorylation and redistribution of connexins and loss of functional coupling (Giaume et al., 1991; Nuriya et al., 2018). This is in agreement with a study showing that α1-NAR signaling can decrease the propagation of [Ca^2+^]_i_ waves through an astrocyte network (Muyderman et al., 1998). In contrast, activation of β-NARs appears to have opposite actions, opening gap junctions and increasing permeability (Giaume et al., 1991; Scemes et al., 2017). This has potentially important consequences on neuronal function, as gap-junction-mediated communication between astrocytes plays a role in the functional and metabolic coupling of astrocytes and neurons (Pannasch and Rouach, 2013; Mayorquin et al., 2018).

Cultured astrocytes change morphology on exposure to NA, adopting a more compact, less branched structure (Bar El et al., 2018), although the NAR mediating this effect remains unknown. Other studies found that β-NAR signaling has the opposite effect, promoting astrocyte stellation in cultures (Vardjan et al., 2014; Koppel et al., 2018) and increasing astrocyte volume in tissue slices. These changes in astrocyte volume will directly impact the size of the extracellular space (Sherpa et al., 2016), impacting on neuronal excitability, by affecting the ability of astrocytes to regulate extracellular neurotransmitter and K^+^ levels (Zhou et al., 2019; Henneberger et al., 2020).

Another function in which the precise role of α1-NAR versus β-NAR signaling in astrocytes remains unclear is the synthesis and secretion of trophic factors, such as brain-derived neurotrophic factor (BDNF) (Juric et al., 2008; Koppel et al., 2018), which are induced by NA.

To summarize, there is substantial evidence suggesting α1-NAR signaling in astrocytes has major functional effects. However, these need to be confirmed *in vivo*, for example, by conditional deletion of α1-NAR from astrocytes (see also section “Noradrenergic Receptors in the CNS” and “Future Strategies to Decipher the Role of NAR Signaling in Heterogenous Astrocyte Populations”). As there seems to be a close relationship to the functional consequences of α1-, α2-, and β-NAR activation, which either elicit opposing or reinforcing effects, a detailed dissection of the role played by each NAR will be necessary to fully understand the consequences of NA signaling on individual astrocytes and, by extension, on CNS circuits.

## α1-NAR Signaling in Astrocytes: Relevance for Neuromodulation

There is abundant literature on the roles of α1-NAR in neuromodulation, which was recently reviewed (Perez, 2020). Most studies highlight a role for α1-NAR in modulating synaptic efficacy and plasticity – and ultimately memory (Perez, 2020). Reports are sometimes contradictory at first glance. For example, one study found that α1-NAR signaling increases long-term potentiation (LTP) at CA3-CA1 synapses in the hippocampus (Izumi and Zorumski, 1999), while another found it increases long-term depression (LTD) at the same synapses (Dyer-Reaves et al., 2019). These discrepancies can likely be attributed to the different experimental protocols and tools used, but what is clear is that α1-NAR signaling affects the plasticity of neuronal circuits, with a common theme being effects on inhibitory transmission (Perez, 2020; and see section “Astrocytes and α1-NAR: A System Tailored to Regulate Inhibition?”). However, a common weakness amongst these studies is again the lack of cell-type specificity in the manipulations used. For example, the majority of studies employ small molecule agonists/antagonists that act on α1-NAR irrespective of the cell type expressing them; some studies use a transgenic mouse model expressing a constitutively active α1-NAR, which potentially does not recapitulate the precise expression profile of the endogenous receptor (see section “Noradrenergic Receptors in the CNS”) (Perez, 2020). In addition to these obvious experimental caveats, most studies on α1-NAR neuromodulation simply (and erroneously in our opinion) assume that NA exerts its actions through direct modulation of neuronal activity. However, astrocytes have a clear role in plasticity mechanisms (Wahis et al., 2021a) and a few (key) studies have made a case for the importance of astrocyte α1-NAR signaling in NA-mediated neuromodulation, most notably in the control of synaptic plasticity.

For example, work in chicks, using metabolic inhibitors to impair astrocyte function, strongly argues for a role of astrocytic α1-NAR signaling in memory consolidation (Gibbs and Bowser, 2010). Another study reports that in the mouse periaqueductal gray, NA promotes arousal through the activation of α1-NAR, which are enriched in astrocytes in this brain region. Interestingly, activating a Gq-coupled Designer Drug Activated by a Designer Receptor (hM3Dq-DREADD), specifically expressed in astrocytes, was sufficient to mimic the positive effects of α1-NAR in this region, reinforcing a role for astrocytes in NA-mediated arousal modulation (Porter-Stransky et al., 2019). Pankratov and Lalo (2015) used several technical approaches to demonstrate that astrocytic α1-NAR signaling is essential for plasticity in the mouse cortex. They found that activation of α1A-NAR, using a subtype-selective agonist, induces the release of both ATP and D-serine in cortical slices. This effect is abolished in mice overexpressing a soluble SNARE protein fragment in astrocytes (dn-SNARE line), which acts in a dominant negative fashion to impair vesicular release of gliotransmitters, by preventing the formation of a productive SNARE core complex necessary for membrane fusion (Scales et al., 2000; Pascual et al., 2005; Jahn and Scheller, 2006; Guček et al., 2016), but note that the astrocyte specificity of dn-SNARE expression is debated (Fujita et al., 2014). They further show that NA induces an increase in the frequency of synaptic currents evoked in pyramidal neurons by purines, an effect abolished both in the dn-SNARE mouse model and when astrocytes are infused with tetanus toxin, a SNARE-specific protease (Holt et al., 2008), which acts to acutely block vesicular release of gliotransmitters. Finally, they found that astrocytic α1-NAR signaling, promoting ATP release in tissue slices, is crucial for LTP induction in visual cortex, which again was blocked by dominant negative SNARE expression or tetanus toxin treatment in astrocytes. Note that earlier work in hypothalamic astrocytes already hinted at a role for astrocytic α1-NAR in eliciting ATP release with subsequent modulation of synaptic activity (Gordon et al., 2005). Monai et al. (2016) demonstrated that transcranial direct current stimulation (tDCS)-induced potentiation of visual responses in cortex is abolished when an antagonist for α1-NAR is applied. Crucially, they found these effects to be dependent on astrocyte Gq signaling, since tDCS is equally ineffective in a transgenic mouse line in which the astrocyte-specific IP_3_R2 receptor is genetically ablated (knocked out: KO), and α1-NAR evoked Ca^2+^ transients are consequently impaired (Srinivasan et al., 2015; Monai et al., 2016). This clearly hints at a core role for astrocytes in conveying the effects of α1-NAR signaling. To date, the strongest evidence for a role of astrocytic α1A-NAR in neuromodulation has been obtained using a recently created *Adra1a*-floxed mouse line, allowing conditional deletion of α1A-NAR in a subset of astrocytes located in the superficial laminae of the spinal cord (Kohro et al., 2020). These astrocytes respond directly to descending noradrenergic projections through α1A-NAR activation. This α1A-NAR-mediated activation of astrocytes is sufficient to potentiate nociceptive inputs, lowering the threshold to mechanical pain in mice, an effect mimicked by astrocyte-specific expression of hM3Dq-DREADDs. This DREADD effect was lost in both IP_3_R2 KO and astrocyte-specific dn-SNARE mice, indicating that both Gq evoked [Ca^2+^]_i_ increases and SNARE-mediated vesicular release from astrocytes were involved. To reveal the signaling pathways mediating astrocyte to neuron communication, the authors blocked several receptors known to be activated by gliotransmitters. While antagonists of P2 purinergic receptors and AMPA receptors did not alter the effects of hM3Dq-DREADD activation on the mechanical pain threshold, 5,7-dichlorokynurenic acid, an antagonist targeting the NMDA receptor co-agonist binding site, did produce effects. This antagonist similarly impaired the nociceptive hypersensitivity induced by the α1-NAR agonist, phenylephrine. This implicates activation of the NMDA receptor co-agonist binding site in α1-NAR-mediated effects on mechanical pain hypersensitivity. Next, the authors identified D-serine, an endogenous (NMDA) receptor co-agonist, as the most likely gliotransmitter involved, as direct injection of D-serine in wild type mice dose-dependently mimicked the effect of α1-NAR activation on mechanical pain threshold. These results indicate the gating of neuronal NMDA receptors, probably through astrocyte-mediated increases in extracellular D-serine, to be the likely mode of astrocyte-to-neuron communication following astrocyte α1A-NAR activation (Figure 1F) (Kohro et al., 2020). This conclusion is in agreement with other studies, which found D-serine levels and neuronal NMDA currents increase in an astrocyte-dependent manner following α1A-NAR activation in mouse cortical slices (Pankratov and Lalo, 2015; Lalo et al., 2018), and that NMDA receptors are implicated in the α1-NAR and astrocyte-dependent potentiation of visually evoked potentials in mice subjected to tDCS (Monai et al., 2016). However, we want to highlight that the study from Kohro et al. (2020) is the only one, to the best of our knowledge, that establishes a clear link between astrocytic α1A-NAR activation and the effects of NA on local circuit function. While other studies suggest a role for α1-NAR signaling in astrocytes, sometimes using multiple lines of evidence (Pankratov and Lalo, 2015), we believe cell-type specific gene ablation approaches are the cleanest way to understand the role of α1-NAR signaling in the CNS. We believe that the recent publication of two *Adra1a*-floxed mouse lines, enabling such experiments (Kohro et al., 2020; Ye et al., 2020), will prove crucial in this endeavor and be a valuable resource for the neuroscience community.

## Astrocytes and α1-NAR : A System Tailored to Regulate Inhibition?

A shared trait among neuromodulators is their effects on inhibitory neurotransmission in the CNS, notably in the cortex (Pacholko et al., 2020). By acting on the inhibitory system, neuromodulators change the gain of specific inputs into cortical circuits, affecting the signal-to-noise ratio of relevant information perceived in the environment (Aston-Jones and Cohen, 2005), shifting the global brain state (Lee and Dan, 2012), and ultimately affecting behavior (Berridge and Waterhouse, 2003). NA-mediated neuromodulation is almost the prototypical example of this model. Indeed, phasic NA release, induced by salient stimuli, will momentarily change the gain of inhibitory circuits (Salgado et al., 2016), which, for instance, will lead to an inhibition of horizontal inputs in the visual cortex, changing the signal-to-noise ratio of sensory inputs (Kobayashi et al., 2000). Crucially, the study by Kobayashi et al. (2000) found these effects to be elicited by α1-NAR activation. There is accumulating evidence that α1-NAR activation increases the activity of GABAergic neurons (directly or indirectly) in multiple CNS regions, including in the hippocampus (Alreja and Liu, 1996; Bergles et al., 1996; Hillman et al., 2009), cortex (Marek and Aghajanian, 1996; Kawaguchi and Shindou, 1998; Kobayashi et al., 2000; Kobayashi, 2007; Lei et al., 2007; Dinh et al., 2009), basolateral amygdala (Braga et al., 2004), spinal cord (Yuan et al., 2009; Seibt and Schlichter, 2015), the bed nucleus of the stria terminalis (Dumont and Williams, 2004), cerebellum (Herold et al., 2005; Hirono and Obata, 2006), nucleus ambiguus (Boychuk et al., 2011), and olfactory bulb (Zimnik et al., 2013). Some of these studies actually used α1-NAR subtype selective antagonists and mostly identified the α1A-NAR as being responsible for mediating responses in GABAergic neurons (Alreja and Liu, 1996; Braga et al., 2004; Hillman et al., 2009; Yuan et al., 2009; Zimnik et al., 2013), with one study reporting effects mediated by α1B-NAR (Marek and Aghajanian, 1996).

From this evidence, it seems that the control of inhibitory transmission through α1-NAR, and probably the α1A-NAR subtype, is a ubiquitous mechanism in the CNS. However, none of these studies could conclusively show which cell type(s) expressed α1A-NAR and were activated by NA. We have already noted that astrocytes are increasingly recognized as the targets of various neuromodulators (Kjaerby et al., 2017; Kastanenka et al., 2020; Pacholko et al., 2020), including NA (O’Donnell et al., 2012), and direct astrocyte activation seems to mimic the shifts in brain state produced by neuromodulators (Poskanzer and Yuste, 2016). The most plausible explanation is that α1A-NAR are expressed by both astrocytes and interneurons, with the functional effects of NA being linked to the concerted activation of both cell types. Indeed, recent single-cell RNA sequencing studies report that both interneurons and astrocytes express *Adra1a* (see section “Noradrenergic Receptors in the CNS”).

Therefore, we hypothesize that α1A-NAR signaling in the CNS might simultaneously activate astrocytes and interneurons, which effectively form a functional unit through which NA exerts its actions. While studies of synaptic regulation typically focus on excitatory (glutamatergic) synapses, there is an increasing number of studies reporting that astrocytes are instrumental in regulating inhibitory (GABAergic) signaling in the CNS (reviewed in Losi et al., 2014; Mederos and Perea, 2019). In particular, three recent studies highlight the importance of astrocytes in the modulation of GABAergic networks, demonstrating that they control the formation of GABAergic synapses through expression of neuronal cell adhesion molecule (NRCAM; Takano et al., 2020), that activation of astrocytic GABA_B_ receptors is crucial for controlling inhibition in cortical circuits and ultimately goal-directed behaviors (Mederos et al., 2021), and that activity of the astrocyte GABA transporter is crucial for the function of circuits in the striatum (Yu et al., 2018). Astrocytes also modulate the activity of GABAergic synapses by releasing the gliotransmitter ATP. Acting through post-synaptic P2X4 receptors, this astrocyte-released ATP decreases the amplitude of both tonic and post-synaptic GABA_A_ currents in neurons (Lalo et al., 2014; Figure 1G). This effect likely plays a key role in regulating astrocytic α1A-NAR-mediated plasticity (Pankratov and Lalo, 2015; and see “α1-NAR Signaling in Astrocytes: Relevance for Neuromodulation”), since P2X4 KO mice have defects in LTP induction (Lalo et al., 2016) and NA-mediated release of ATP by astrocytes has been implicated in experience-dependent metaplasticity (Lalo et al., 2018).

However, the interactions between astrocytes and GABAergic neurons show additional layers of complexity. First, and somewhat counter-intuitively, astrocytes can transform inhibitory signals into excitatory ones in the hippocampus, with astrocytic GABA_B_ receptor activation inducing glutamate release from astrocytes, which potentiates synapses by acting on pre-synaptic metabotropic glutamate receptors (mGluR) (Perea et al., 2016; Mederos et al., 2021). Another study reports that increased activity of the astrocytic GABA transporter, GAT3, following repetitive firing of neuropeptide Y (NPY) positive GABAergic interneurons, elevates [Ca^2+^]_i_ in astrocytes inducing glutamate release, which in turn acts on neuronal mGluR_1_ to modify the spiking patterns of NPY expressing interneurons (Deemyad et al., 2018), in effect creating a feedback loop. Second, a single astrocyte can detect different levels of GABAergic activity and respond by releasing distinct gliotransmitters, with different and opposing effects on synaptic activity (Covelo and Araque, 2018). Activation of astrocytic GABA_B_ receptors, in response to low levels of neuronal activity, potentiates excitatory transmission through a mGluR_1_-based mechanism. However, high levels of interneuron activity over prolonged periods eventually leads to synaptic depression, mediated by activation of neuronal A_1_ receptors, probably following astrocytic release of ATP with subsequent degradation into adenosine. Moreover, this biphasic modulation could be induced by artificially manipulating the [Ca^2+^]_i_ in a single astrocyte. This notion of biphasic modulation of synaptic transmission by astrocytes is further reinforced by studies demonstrating that ATP and D-serine, which are known to be released by astrocytes following α1A-NAR activation (Pankratov and Lalo, 2015), also have opposing actions on synaptic activity. D-serine (and glutamate) gate the activation of NMDA receptors, increasing overall excitability and boosting LTP; in contrast, ATP activates neuronal P2X receptors depressing the extent of LTP induction. Mechanistically, it appears this astrocyte-dependent activation of P2X receptors leads to a down-regulation of NMDA receptors at excitatory synapses (Lalo et al., 2016, 2018).

Taken together, these studies reinforce the concept of lateral regulation of synaptic transmission by astrocytes. According to this concept, one astrocyte receiving inputs from an inhibitory interneuron can also modulate an excitatory synapse, and vice versa (Figure 1H, blue and red arrows). This phenomenon can be extended to incorporate distant synapses, with the activation of one astrocyte being relayed to other cells through either paracrine or gap-junction-based intercellular communication (Figure 1I; Covelo and Araque, 2016). These numerous mechanisms exemplify the complexity of astrocyte-neuron interactions, and show that astrocytes can modulate circuits in multiple ways depending on the patterns of activity they detect at synapses, as well as the presence of neuromodulators. For interested readers, these last issues are discussed in depth in a parallel publication in this edition of Frontiers in Cellular Neuroscience (Caudal et al., 2020).

We believe that the concomitant activation of astrocytes and interneurons by NA through α1-NAR might harness the mechanisms we have discussed in this section to convey (at least some of) the effects of NA. Other signaling mechanisms, namely the synergistic effects of synaptic signaling and cellular ‘priming’ by NA in astrocytes (see section “α1-NAR Triggered Calcium Signaling in Astrocytes: A Switch for Signal Integration?”), are also likely to participate in the mechanisms underlying astrocyte-interneuron crosstalk. However, the relative contributions of these two cell types to the effects elicited by NA remains to be elucidated. This will, no doubt, require precise cell-type (and perhaps even cell subtype) specific manipulation of NA signaling, coupled to readouts of functional effects (also see sections “NA Signaling Through Astrocytes: The Impact of Cell Heterogeneity” and “Future Strategies to Decipher the Role of NAR Signaling in Heterogenous Astrocyte Populations”).

## Astrocyte–Neuron Interactions Following α1-NAR Signaling: Positive Feedback Mechanisms for NA Release?

The complexity of astrocyte-neuron interactions underlying NA signaling might have a further layer of complexity. Indeed, Mather et al. (2016) have proposed an interesting model, which they term “Glutamate Amplifies Noradrenergic Effects” (GANE). In this model, NA release is amplified at sites of high activity, by the action of synaptically released glutamate. This glutamate accumulates and activates receptors expressed on NA varicosities located in proximity to the active synapse, evoking further release of NA from active LC axons. Elevated concentrations of NA then activate post-synaptic β-NAR, increasing synaptic weight. Astrocytes can be factored into this model in several ways. First, astrocytes may respond to the presence of either, or both, released neurotransmitters and NA by releasing glutamate and D-serine, further boosting NA release (Figure 1J). Second, as astrocytes activated by GABAergic signaling can release glutamate, they may also potentiate activity at active GABAergic synapses by increasing NA release from LC varicosities in close proximity (Figure 1K). Third, high local concentrations of NA may activate astrocytic β-NAR, leading to increased production and secretion of L-lactate, which is known to increase the firing rate of LC axons, leading to further NA release (Tang et al., 2014). Finally, astrocytes function as “signal integrators” (see section “α1-NAR Triggered Calcium Signaling in Astrocytes: A Switch for Signal Integration?”): in this case, α1A-NAR activation in astrocytes is likely to be enhanced by GABA and somatostatin release from α1A-NAR expressing interneurons (Marin et al., 1993; Hillman et al., 2009; Mariotti et al., 2018). This will lead to increased astrocyte [Ca^2+^]_i_ and the subsequent release of glutamate, which will itself lead to increased NA release. It is interesting to speculate that the astrocytic release of ATP, associated with high levels of interneuron activity (Covelo and Araque, 2018), might serve as a brake in this system, acting to limit (or inhibit) release of NA from varicosities by activating A_1_ receptors following extracellular degradation to adenosine. Similarly, astrocyte-released ATP, signaling through neuronal P2X receptors, may lead to the down-regulation of NMDA-mediated currents (Lalo et al., 2016) limiting neuronal excitability.

Many unanswered (and exciting) questions remain in this area. For example, what are the relative roles of astrocyte-interneuron interactions in mediating the effects of NA on circuits? One area of increasing importance to the astrocyte field is the issue of cellular heterogeneity (see section “NA Signaling Through Astrocytes: The Impact of Cell Heterogeneity”): it will be crucial for our understanding of CNS function to know if astrocytes express or respond to α1-NAR activation in a homogeneous or heterogeneous function and how this impacts circuit activity.

## NA Signaling Through Astrocytes: The Impact of Cell Heterogeneity

Analysis of single cell transcriptome data shows that the *Adra1a* transcript is one of the few that is detectable in all cortical astrocytes (see section “Noradrenergic Receptors in the CNS”). This finding corroborates the results of several functional studies, which have found that application of phenylephrine, an α1-NAR agonist, reliably evokes [Ca^2+^]_i_ responses in the majority (if not all) cortical astrocytes (Ding et al., 2013; Srinivasan et al., 2015, 2016). However, in a recent study from our own lab, we found that the dynamics of the α1-NAR evoked Ca^2+^ transients differ between cortical layers (Batiuk et al., 2020). Astrocytes in different cortical layers have also been shown to have different morphologies (Lanjakornsiripan et al., 2018) and molecular profiles (Bayraktar et al., 2020), which we hypothesize drive differential [Ca^2+^]_i_ signaling (Semyanov et al., 2020) and functional outputs (Bagur and Hajnóczky, 2017). We regard this as interesting because NA is known to have layer-specific effects on inhibitory transmission in the cortex (Salgado et al., 2016), suggesting that α1A-NAR signaling in otherwise heterogeneous astrocyte populations might produce different downstream effects on neuronal function(s). Yet further evidence supporting a role for functionally specialized cortical astrocyte subtypes is work demonstrating that activation of astrocytes specifically in supragranular layers is important for neuronal reactivation, following loss of sensory inputs into the visual cortex after monocular enucleation (Hennes et al., 2020). As there is increasing evidence that astrocytes show anatomical, molecular and physiological specialization across brain regions (reviewed in Bayraktar et al., 2015; Farmer and Murai, 2017; Dallérac et al., 2018; Khakh and Deneen, 2019; Pestana et al., 2020), we expect astrocyte heterogeneity will emerge as a major factor contributing to the complex, differential actions of NA in the CNS (O’Donnell et al., 2012; Perez, 2020). In fact, α1-NAR signaling seems a uniform property of astrocytes, with evidence for phenylephrine-evoked [Ca^2+^]_i_ transients in multiple brain regions, including in striatum (Yu et al., 2018), hippocampus (Duffy and MacVicar, 1995), cerebellum (Kulik et al., 1999), ventrolateral medulla (Schnell et al., 2016), hypothalamus (Gordon et al., 2005), and spinal cord (Kohro et al., 2020). However, consistent with our hypothesis, α1A-NAR signaling appears to elicit differing Ca^2+^ responses in regionally distinct cells, with Pham et al. (2020) uncovering differences between hypothalamic and cortical astrocytes. Unfortunately, the complex molecular signatures of astrocyte subtypes identified to date means that new genetic tools will likely be required to fully probe the functional roles of α1A-NAR expressing subtypes (see section “Future Strategies to Decipher the Role of NAR Signaling in Heterogenous Astrocyte Populations”).

## Future Strategies to Decipher the Role of Nar Signaling in Heterogenous Astrocyte Populations

In our opinion, the ability to target functional astrocyte subtypes will likely need the use of intersectional genetics. This typically uses simultaneous activation of multiple promoters to achieve selective transgene expression in the desired cell subtype. An obvious example is the split Cre system (Hirrlinger et al., 2009), which could, for example, be used in combination with *Adra1a*-floxed mice to delete α1A-NAR in specific astrocyte subtypes (Kohro et al., 2020; Ye et al., 2020), or induce expression of a fluorescent protein (or genetically encoded sensor) in cells using existing mouse lines (such as GCaMP6f in the Ai95D line^2^).

Obtaining the genetic fingerprint of spatially resolved and functionally distinct cell populations remains difficult. At the time of publication, spatial transcriptomic approaches, based on *in situ* sequencing, lack single cell resolution (Ståhl et al., 2016; Ortiz et al., 2020). Although somewhat cumbersome, it is, however, possible to map back unique cell subtypes, identified using single cell RNA-seq approaches, to their anatomical positions, using highly multiplexed *in situ* hybridization techniques, as we demonstrated in recent publications (Batiuk et al., 2020; Bayraktar et al., 2020). Unique transcripts, defining cell subtypes, can then theoretically be used to identify promoters for use in intersectional approaches. It should be noted, however, that promoter mapping is notoriously difficult, and in some cases even 500 bp of sequence is sufficient to completely change the pattern of gene expression (Miller et al., 2019). Given the need to screen multiple promoter fragments, to ensure the endogenous gene expression profile is faithfully recapitulated, we anticipate the manufacture of transgenic mouse lines will prove too laborious and costly for many questions of interest. The use of viral vectors offers an attractive alternative (Beckervordersandforth et al., 2010, 2014), although these typically suffer from limited capacity (Luo et al., 2018) and may not be able to incorporate the full regulatory sequence required to recapitulate the endogenous pattern of gene expression (Regan et al., 2007). To overcome these limitations, our lab is developing a PiggyBac-based system, misPiggy, for flexible gene expression in the CNS, based on *in utero* electroporation (Slezak et al., 2018: pre-print). This simple, one plasmid solution can be easily used to perform rapid analysis of putative promoter sequences based on fluorescent protein expression (Wang et al., 2007), while its large cargo capacity allows the incorporation of multiple independent expression modules, making it ideal for intersectional genetics. We expect this system will allow us to make substantial progress in deciphering the contribution of unique astrocyte subtypes to NA-mediated control of local circuit function.

Understanding the biology of α1-NAR signaling in astrocytes (as well as that of other NAR types) will benefit from further advances in the development of genetically encoded biosensors. Changes in astrocyte [Ca^2+^]_i_ are still considered the best measure of astrocyte activity (Verkhratsky and Nedergaard, 2018; Caudal et al., 2020). New genetically encoded calcium indicators (GECIs), such as the jGCaMP7 series, are under intense development, and benefit from improved properties, such as signal-to-noise ratio and steady-state brightness (Dana et al., 2019). Other developments include red-shifted GECIs (Dana et al., 2016), which allow simultaneous dual color (green-red) imaging in astrocytes and neurons (including LC axons) (Bindocci et al., 2017; Oe et al., 2020). Of importance, the use of red-shifted GECIs can also be combined with optogenetic tools, allowing precise cell stimulation (Forli et al., 2018). However, as discussed earlier (see section “α1-NAR Signaling: Effects on Astrocyte Function”), Ca^2+^ is not the only important secondary messenger, or metabolite, whose level changes following astrocyte stimulation with NA. Examples include cAMP, whose levels can be measured using the red-shifted sensor PinkFlamindo (Oe et al., 2020), as well as lactate, which can be measured using the Laconic system (San Martín et al., 2013; Mächler et al., 2016; Zuend et al., 2020). Furthermore, genetically encoded sensors exist for the measurement of various neurotransmitters and neuromodulators (including NA; Patriarchi et al., 2018; Feng et al., 2019; Oe et al., 2020 and further reviewed in Leopold et al., 2019). New genetically encoded sensors are constantly being developed (Leopold et al., 2019) and will undoubtedly bring new insights in the mechanisms of NA release. When these are combined with sensors for cell-type specific second messenger detection, we will be able to dissect out the mechanisms by which NA exerts its actions across CNS cell types. Measurements of ionic species beyond calcium is also possible, with genetically encoded fluorescent sensors for K^+^ (Shen et al., 2019), H^+^ and Cl^–^ (Germond et al., 2016) currently available.

Finally, to understand the role(s) of astrocytes in neuromodulation and beyond, there is a clear need for tools allowing precise and reproducible cell activation. Optogenetic control of astrocytes, using different channelrhodopsin variants or proton pumps, has already been attempted (Figueiredo et al., 2014; Xie et al., 2015; Cho et al., 2016). However, the mechanism(s) through which a light-sensitive cation channel can evoke [Ca^2+^]_i_ transients in astrocytes remain unclear (Figueiredo et al., 2014; Octeau et al., 2019); in fact, channelrhodopsins have recently been criticized as having unwanted effects, such as promoting increases in [K^+^]_e_ (Octeau et al., 2019), which lead to secondary increases in neuronal activity that compound the interpretation of experimental data. It is now becoming clear that a much better approach to optical control of astrocyte function is the use of light-gated GPCRs, such as the Gq-GPCR Optoα1AR. This chimeric receptor is actually based on the intracellular amino acid sequence of the α1A-NAR (Airan et al., 2009), and has recently been used to elicit Gq signaling in astrocytes (Adamsky et al., 2018; Iwai et al., 2020: pre-print). An example of a naturally occurring protein that has been repurposed is the Gq-coupled photopigment melanopsin, which has been used to study the role of astrocytes in GABAergic transmission (Mederos et al., 2019, 2021). Optogenetics has the important advantage of allowing fine spatial and temporal control of cell activation, which is harder to attain when using chemogenetic (DREADD-based) approaches, which are limited by the pharmacodynamics of their synthetic agonists. However, DREADDs have the advantage of allowing simultaneous activation of cells across a broad tissue area, without the need for invasive implantation of optical devices. As such, they are being increasingly used in neuroscience research (Roth, 2016), with particular success in helping elucidate the roles played by astrocytes in the control of CNS circuits (Adamsky et al., 2018; Hennes et al., 2020; Erickson et al., 2021).

We believe that the use of these tools in innovative and imaginative combinations will undoubtedly bring new insights into astrocyte functions in the CNS, including (and importantly) their role(s) in NAR signaling.

## Conclusion

With this review, we aimed to provide an overview of the current state of the art on α1-NAR signaling in astrocytes, and how this relates to NA-mediated neuromodulation. We believe that α1-NAR signaling is a major signaling pathway in astrocytes, which switches the cell into a more sensitive state, effectively ‘priming’ it to integrate signals originating from other cells in the immediate environment. We advance the hypothesis that both GABAergic interneurons and astrocytes are involved in α1A-NAR signaling, with the two cell types forming a functional unit, through which NA exerts its neuromodulatory effects. We argue that these issues will need to be considered in light of the fact that astrocytes show considerable heterogeneity in morphology, transcriptome/proteome and physiology, and discuss tools to potentially solve these issues. For brevity, we deliberately chose not to discuss aspects of astrocyte α1-NAR signaling that are potentially involved in response to developmental deficits (D’Adamo et al., 2021), injury (Vardjan et al., 2016) or disease (Zorec et al., 2018). However, given the presence of astrocytes throughout the CNS and their ubiquitous responses to NA, it is likely that aberrant NA-mediated Ca^2+^ signaling plays a central role in many conditions. Therefore, understanding α1-NAR signaling and how it modulates astrocyte functions (including interactions with other cell types) will not only lead to a better understanding of CNS function in the healthy brain, it will likely provide insights into the mechanisms underlying injury and disease.

## Author Contributions

JW and MGH participated equally in the writing of this review. Both authors contributed to the article and approved the submitted version.

## Conflict of Interest

The authors declare that the research was conducted in the absence of any commercial or financial relationships that could be construed as a potential conflict of interest.

## Footnotes

1 https://www.proteinatlas.org/ENSG00000120907-ADRA1A/tissue/cerebral+cortex#imid_8462679

2 https://www.jax.org/strain/024105
